# The Natural Anthraquinone Parietin Inactivates *Candida tropicalis* Biofilm by Photodynamic Mechanisms

**DOI:** 10.3390/pharmaceutics17050548

**Published:** 2025-04-23

**Authors:** Juliana Marioni, Bianca C. Romero, Ma. Laura Mugas, Florencia Martinez, Tomas I. Gómez, Jesús M. N. Morales, Brenda S. Konigheim, Claudio D. Borsarelli, Susana C. Nuñez-Montoya

**Affiliations:** 1 Departamento de Ciencias Farmacéuticas, Facultad de Ciencias Químicas, Universidad Nacional de Córdoba, Haya de la Torre y Medina Allende, Ciudad Universitaria, Córdoba X5000HUA, Argentina; juliana.marioni@unc.edu.ar (J.M.); bianca.romero@unc.edu.ar (B.C.R.); tomasigomez0@gmail.com (T.I.G.); 2 Instituto de Bionanotecnología del NOA (INBIONATEC), Universidad Nacional de Santiago del Estero—CONICET, RN9, Km 1125, Santiago del Estero G4206XCP, Argentina; jesusmarcelom@gmail.com (J.M.N.M.); cdborsarelli@gmail.com (C.D.B.); 3 CONICET, Unidad de Investigación y Desarrollo en Tecnología Farmacéutica (UNITEFA), Haya de la Torre y Medina Allende, Ciudad Universitaria, Córdoba X5000HUA, Argentina; 4 Centro de Investigaciones sobre Porfirinas y Porfirias (CIPYP), CONICET and Hospital de Clínicas José de San Martín, Universidad de Buenos Aires, Córdoba 2351 1er subsuelo, Ciudad de Buenos Aires 1120AAF, Argentina; 5 Instituto de Virología “Dr. J. M. Vanella”, Facultad de Ciencias Médicas, Universidad Nacional de Córdoba, Córdoba X5000HUA, Argentina; florencia.martinez@unc.edu.ar (F.M.); brenda.konigheim@unc.edu.ar (B.S.K.); 6 Consejo Nacional de Investigaciones Científicas y Técnicas (CONICET), Córdoba X5000HUA, Argentina

**Keywords:** natural anthraquinone, superoxide anion, singlet oxygen, *Candida* biofilm, photodynamic therapy, photoinactivation, reactive oxygen species, oxidative stress

## Abstract

**Background/Objectives**: Parietin (PTN), a blue-light absorbing pigment from *Teloschistes* spp. lichens, exhibit photosensitizing properties via Type I (superoxide anion, O_2_^•−^) and Type II (singlet oxygen, ^1^O_2_) mechanisms, inactivating bacteria in vitro after photoexcitation. We evaluate the in vitro antifungal activity of PTN against *Candida tropicalis* biofilms under actinic irradiation, its role in O_2_^•−^ and ^1^O_2_ production, and the cellular stress response. **Methods**: Minimum inhibitory concentration (MIC) of PTN was determined in *C. tropicalis* NCPF 3111 under dark and actinic light conditions. Biofilm susceptibility was assessed at MIC/2, MIC, MICx2, MICx4, and MICx6 in the same conditions, and viability was measured by colony-forming units. Photodynamic mechanisms were examined using Tiron (O_2_^•−^ scavenger) or sodium azide (^1^O_2_ quencher). O_2_^•−^ production was measured by the nitro-blue tetrazolium (NBT) reduction and nitric oxide (NO) generation by Griess assay. Total antioxidant capacity was studied by FRAP (Ferrous Reduction Antioxidant Potency) assay and superoxide dismutase (SOD) activity by NBT assay. **Results**: Photoexcitation of PTN reduced *C. tropicalis* biofilm viability by four logs at MICx2. Sodium azide partially reversed the effect, whereas Tiron fully inhibited it, indicating the critical role of O_2_^•−^. PTN also increased O_2_^•−^ and NO levels, enhancing SOD activity and FRAP. However, this antioxidant response was insufficient to prevent biofilm photoinactivation. **Conclusions**: Photoinactivation of *C. tropicalis* biofilms by PTN is primarily mediated by O_2_^•−^, with a minor contribution from ^1^O_2_ and an imbalance in NO levels. These findings suggest PTN is a promising photosensitizer for antifungal photodynamic therapy.

## 1. Introduction

Antimicrobial (ATM) resistance is currently a global problem that hinders the treatment of common infections, increasing the risk of serious and even fatal complications. The prolonged duration of treatment has ramifications for other domains, including the escalation of healthcare costs, both for patients and healthcare systems, due to the use of more complex and expensive treatments. Furthermore, ATM resistance reduces the safety of some medical procedures (surgeries, transplants, cancer treatments) by increasing the risk of resistant infections. This is compounded by the rapid spread of infection, which makes it difficult to control and increases the risk of epidemic outbreaks [1].

Invasive fungal infections represent a significant challenge in the clinical setting, especially those caused by species of the genus *Candida*, an opportunistic fungus responsible for most hospital-acquired infections with a high morbidity and mortality rate. The ability of *Candida* spp. to exhibit ATM resistance has been linked to their capacity to form biofilms, which are distinct in that they are formed by both morphotypes—the yeast and hyphal forms—that intertwine to create a complex three-dimensional structure. This structure affords biofilms greater resistance to traditional antifungals than their planktonic counterparts. Within this group, *Candida tropicalis* stands out for its ability to form biofilms that are highly resistant to conventional treatments, which makes eradication difficult and favors the persistence of infections in immunocompromised patients [2,3].

In the face of ATM resistance, a multitude of strategies have been proposed. Among these, antimicrobial photodynamic inactivation (aPDI) has emerged as a promising approach. This strategy involves the generation of reactive oxygen species (ROS) following the excitation of a photosensitizer (PS) with light of a specific wavelength. This approach has proven to be effective against various pathogenic bacteria, parasites, viruses, and fungi, controlling toxicity on human cells by matching the three necessary factors (PS at a non-active concentration in darkness, light, and oxygen) in the area to be treated. The triplet excited state of the PS (^3^PS*) may react with molecular oxygen (^3^O_2_) by two mechanisms: a charge transfer reaction to generate superoxide anion radical (O_2_^•−^), or an energy transfer reaction generating singlet molecular oxygen (^1^O_2_), also called Type I and Type II processes, respectively. These species thus generated and the species derived from them by secondary reactions (hydrogen peroxide: H_2_O_2_; and hydroxyl radical: HO^•^), are ultimately those that oxidize the biological molecules in their direct environment (proteins, lipids, DNA), triggering cellular oxidative stress that eventually leads to the death of the microorganisms, which translates into regression of the infection [4,5].

In the search for new therapeutic agents, natural products have become of interest due to their structural diversity and bioactive potential [6]. Among them, lichens are an important source of secondary metabolites with antimicrobial, antioxidant, and cytotoxic properties [7,8,9,10,11,12]. These symbiotic associations between fungi and algae or cyanobacteria produce a wide variety of terpenoid and phenolic derivatives, including among the latter anthraquinones (AQs), many of which have demonstrated activity against pathogenic bacteria and fungi [7].

AQs are a family of compounds found in higher plants and lichens with photoactive properties and potential antimicrobial activity. Some AQ derivatives have shown the ability to generate ROS under irradiation, suggesting their possible application in the inactivation of pathogens. However, the mechanism of action of these molecules and their impact on the viability and structure of fungal biofilms still require further investigation [13,14].

**Figure 1:**
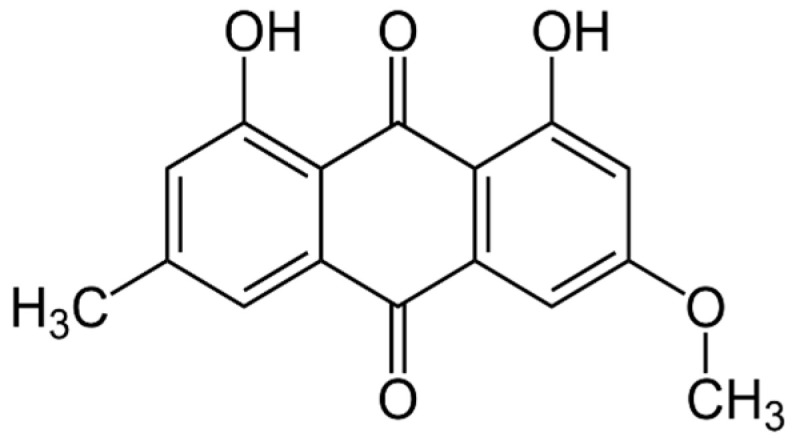
Chemical structure of parietin (1,8-dihydroxy-3-methoxy-6-methyl-9,10-anthraquinone, PTN).

Parietin (PTN), 1,8-dihydroxy-3-methoxy-6-methyl-9,10-anthraquinone (Figure 1), is found as orange crystals in the upper crust of lichens of the *Teloschistaceae* family, acting as an UV-B photoprotective pigment [15]. However, we have previously shown that PTN diluted in organic solvents such as chloroform is an efficient PS of ^1^O_2_ with a high quantum yield (Φ_Δ_ = 0.69) as well as an O_2_^•−^ photogenerator in aqueous media [16].

In this work, the in vitro antifungal and antibiofilm activity of PTN was evaluated both in dark conditions and under irradiation. The aim is to determine its potential as a PS against *C. tropicalis* biofilms. The involvement of Type I and Type II photosensitizing mechanisms in the generation of ROS was analyzed; and because of this, the generation of reactive nitrogen intermediates (RNI) was also assessed, since these species can be formed as a secondary process of deactivation of O_2_^•−^. The superoxide dismutase enzyme (SOD) activity and the total antioxidant capacity of the biological system (employing FRAP, Ferrous-Reducing Antioxidant Potency assay) were also evaluated, to establish whether oxidative or nitrosative stress would be responsible for the observed biological effect [17,18,19].

The results of this study indicate that the photoinactivation of *Candida tropicalis* biofilms by PTN is predominantly driven by the O_2_^•−^ generation (Type I mechanism). Although the ^1^O_2_ (Type II mechanism) also plays a role in this process, its contribution is considerably smaller compared to O_2_^•−^. Furthermore, the study revealed an imbalance in NO levels, which likely exacerbates the overall cellular stress response, contributing to the effectiveness of the photodynamic treatment. Given these findings, PTN shows strong potential as an effective PS in antifungal photodynamic therapy (aPDT), offering a promising therapeutic approach for treating fungal biofilm-related infections.

## 2. Materials and Methods

### 2.1. Reagents and Solvents

Sabouraud dextrose broth (SDB) and Sabouraud dextrose agar (SDA) were obtained from Britania, CABA, Argentina. Sodium nitrite (NaNO_2_), and ferrous sulfate (FeSO_4_) were acquired from Cicarelli, Sta. Fe, Argentina. Sodium hydroxide (NaOH, Biopack, Zárate, Argentina) and Amphotericin B (AmB, Richet, Tres Arroyos, Argentina), phosphate-buffered saline (PBS) were also acquired. Roswell Park Memorial Institute 1640 (RPMI), morpholine propane sulfonic acid (MOPS), sodium azide (NaN_3_), Tiron, nitro-blue tetrazolium (NBT), methionine, and riboflavin were purchased from Sigma, St. Louis, MO, USA. Water and distilled solvents (Sintorgan, Buenos Aires, Argentina) were used.

### 2.2. Yeast Strain and Growth Conditions

A standard strain was used: *Candida tropicalis* NCPF 3111 (National Collection of Pathogenic Fungi, Bristol, UK). It was conserved and reactivated according to Clinical and Laboratory Standards Institute guidelines (CLSI, 2002) [20]. Sabouraud dextrose broth (SDB) was used as a growth medium at 37 °C.

### 2.3. Natural Photosensitizer Tested

Parietin (PTN) (Figure 1) was obtained from the lichen *Teloschistes nodulifer* (Nyl.) Hillman (Teloschistáceas, identified as CORDC00005354, Museo Botánico de Córdoba, Universidad Nacional Córdoba). In the purification and identification process, the methodology previously developed was followed to obtain a purity of 95%, according to HPLC [21,22]. A hydroalcoholic solution of PTN (3.5 mM) was used as a stock solution, containing 1% ethanol (EtOH). The tested concentrations were prepared by diluting the stock with a culture medium, depending on whether the assay was carried out on planktonic yeasts or biofilm.

### 2.4. Irradiation System

Photoinactivation assays were carried out using an actinic Phillips 20W lamp (380 ± 480 nm, 0.65 mWcm^−2^, Madrid, Spain) with an emission maximum at 420 nm, which was placed inside a black box at 20 cm above the samples [22].

### 2.5. Photoactive Minimum Inhibitory Concentration

The lowest concentration of the compound that inhibits the growth of the planktonic form of *C. tropicalis* was determined. The protocol described by CLSI (2008) [23] was applied to determine the minimum inhibitory concentration (MIC), adapted to be carried out under dark and irradiated conditions [24]. This allows the establishment of the photoactivated MIC (pMIC) in comparison to the MIC in darkness, suggesting the photodynamic potential of the PS. The assay was carried out on 96-well microplates (Greiner Bio-One, Frickenhausen, Germany) containing the following culture medium: RPMI 1640 with glutamine and 0.2% glucose, without sodium bicarbonate, buffered with 0.164 M MOPS and adjusting the pH to 7 with 1N NaOH. PTN solutions (100 μL) were added to each well following the additive serial double dilution method and twelve concentrations between 0.24 and 500 µg/mL were tested in triplicate. AmB was the positive control (+C), used at its MIC. The yeast suspension (100 μL) was then added to each well with a final concentration of 0.5 × 10^3^–2.5 × 10^3^ CFU/mL. One set of these concentrations was irradiated for 15 min at room temperature, whereas another set was shielded from light. Immediately after this time, both sets were incubated at 37 °C for 48 h. Finally, the optical density (OD) of each well was measured at 490 or 530 nm on a microplate reader (Tecan Sunrise Model, TECAN, Grödig, Austria). RPMI and yeast suspension with 1% EtOH, both without PTN, were embraced as controls (in triplicate) in both experimental conditions.

In addition, a colony-forming unit (CFU) count assay was performed. A sample (100 µL) was taken from each well, which was diluted 1:10 in PBS until final dilution 1:1,000,000. Subsequently, each suspension (100 μL) was seeded on Sabouraud dextrose agar (SDA) plates and incubated for 48 h at 37 °C. Before incubation, the CFU were counted to establish the minimum fungicidal concentration (MFC), defined as the lowest concentration of a compound that kills 99.9% of a fungal inoculum.

### 2.6. Photoinactivation Biofilm Procedures

Biofilms were formed in flat-bottomed 96-well microplates (Greiner Bio-One, Frickenhausen, Germany), following an adaptation from the method of O’Toole & Kolter [22]. Once a 48 h biofilm formation in SDB was achieved, the microplates were rinsed twice with PBS (200 µL × 2) at pH = 7 to remove non-adherent cells. PTN was tested on *C. tropicalis* biofilms at five concentrations in triplicate: pMIC/2, pMIC, pMICx2, pMICx4, and pMICx6, under darkness and irradiation conditions. SDB alone and SDB with 1% EtOH were included as negative controls. AmB (+C) was used as a positive control at MIC. After treatment, microplates were incubated at 37 °C for 48 h. The supernatant was replaced by PBS (100 µL per well) and sonicated (40 kHz, 60 s in Codyson CD4831, Shenzhen, China). The biofilm of each well was removed and serially diluted with PBS. Each dilution was plated (10 μL) on the SDA plate in triplicate, and the number of CFUs formed after 48 h incubation at 37 °C was counted. The counting of CFU/mL was log-transformed.

### 2.7. Mechanism Action Studies

To determine the mechanism of photosensitizing action, the scavenging effect of sodium azide on singlet oxygen (^1^O_2_ quencher) or Tiron on superoxide anion (O_2_^•−^ scavenger) was evaluated in PTN-treated and untreated biofilm, both in the presence and absence of light [25]. Quencher/scavenger and PTN solutions were added on a dense biofilm (48 h) at the same time, so that the final concentration of each was 200 μM and the PTN concentrations were pMIC/2, pMIC, pMICx2, pMICx4, pMICx6. After darkness and irradiation treatment (15 min), the procedure continued as described above for CFU quantification.

Each supernatant of both microplates (dark and light) was extracted to assess the production of O_2_^•−^, reactive nitrogen intermediates (RNI), and the activation of the antioxidant system: SOD and total non-enzymatic system using the FRAP assay.

Production of O_2_^•−^ was determined by the nitro-blue tetrazolium (NBT) reduction method [26]. Blue diformazan formation is proportional to the generated O_2_^•−^ in biofilms and its OD was measured at 540 nm on the same microplate reader. Results were expressed as OD_540nm_/CFU (Superoxide anion/CFU.mL^−1^) [22].

RNI generation was evaluated as nitrite formation, by using the Griess reaction and a calibration curve of NaNO_2_ as the standard [27]. OD was measured spectrophotometrically at 540 nm [22] and results were expressed as the ratio between nitrite concentration values/CFU (RNI/CFU.mL^−1^).

SOD activity was evaluated by the ability of this enzyme to inhibit NBT reduction in the presence of O_2_^•−^, generated by the photostimulation of riboflavin in the presence of oxygen and an electron donor (methionine). Results were expressed as SOD activation (%SOD/CFU.mL^−1^) [22,28].

FRAP assay was used following the methodology described by Benzie & Strain (1996) [29]. The absorbance was measured at 593 nm and results were expressed as the Fe^2+^ concentration values/CFU (FRAP/CFU.mL^−1^) by using a FeSO_4_ calibration curve [22].

### 2.8. Statistical Analysis

All assays were made in triplicate of three independent experiments. Data were expressed as means ± standard deviation. A *p*-value < 0.05 was considered statistically significant, obtained by the t-Student–Newman–Keuls test for multiple comparisons. The symbol * denotes statistical significance at *p* < 0.05 when compared to untreated biofilms and # indicates statistical significance at *p* < 0.05 when darkness and irradiation were compared.

### 2.9. Declaration of Generative AI–AI-Assisted Technologies

During the preparation of this work, the author(s) used ChatGPT (GPT-4 version) to improve the redaction and grammar of English. After using this tool/service, the authors reviewed and edited the content as needed and assume full responsibility for the publication’s content.

## 3. Results

### 3.1. Photoactive Minimum Inhibitory Concentration (pMIC)

**Figure 2:**
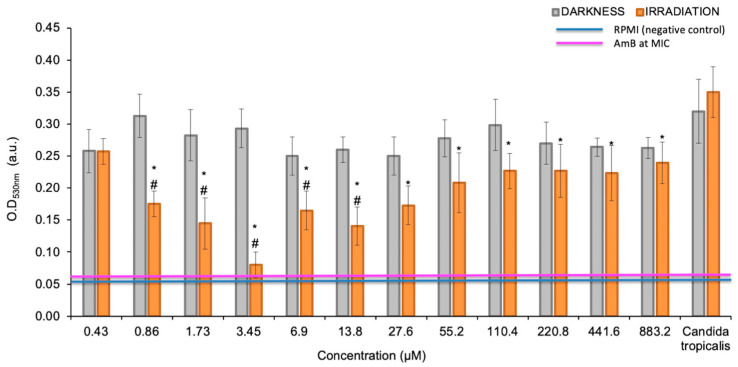
MIC determination of parietin (PTN) under darkness and irradiation conditions. Optical density values obtained for planktonic cells at 530 nm, previously treated with different concentrations of PTN. * *p* < 0.05 with respect to *C. tropicalis*. # *p* < 0.05 darkness vs. irradiation.

PTN produced most significant growth inhibition of planktonic yeasts of *C. tropicalis* at 0.98 μg/mL (≈3.45 µM) under irradiation conditions, as defined as the photoactive MIC (Figure 2). Furthermore, these values are below the cytotoxic concentration of PTN, since the cell viability of Vero cells was 80% and 60% at 100 µg/mL, under darkness and light, respectively [16]. In contrast, in the dark, PTN only produces 20% growth inhibition; therefore, it is not possible to determine its MIC.

### 3.2. PTN Antimicrobial Photodynamic Therapy (APDT)

**Figure 3:**
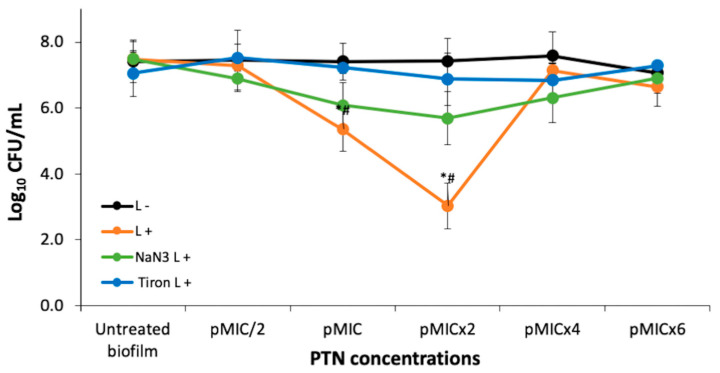
Photoinactivation studies and photodynamic mechanism of *C. tropicalis* biofilms with parietin at five concentrations, using pMIC (0.98 μg/mL ≈ 3.45 μM) as reference and actinic irradiation (L+) vs. darkness (L−). * *p* < 0.05 with respect to *C. tropicalis*. # *p* < 0.05 darkness vs. irradiation.

The antifungal activity of PTN was also tested on *C. tropicalis* biofilms at five concentrations taking as reference the pMIC value (0.98 μg/mL ≈ 3.45 μM), obtained in yeast suspensions. CFU were not affected by light conditions. Regarding the antibiofilm activity of PTN on *C. tropicalis* (Figure 3), we can state that it had no effect at the tested concentrations in the dark. On the other hand, when PTN was photostimulated, it could be observed that the photodynamic effect was concentration-dependent, starting at the pMIC and the most active concentration being pMICx2 (≈2 μg/mL), as it produced a 4-log CFU reduction. At the highest concentrations tested, no photoinactivation was observed, probably because molecular aggregates of PTN in aqueous media increase the prompt non-radiative deactivation of the singlet excited state, avoiding the generation of the ^3^PTN* and therefore the photogeneration of ROS [25].

#### 3.2.1. Photodynamic Mechanism on Analysis After PTN-APDT

To clarify the photoinactivation mechanism, the amount of photogenerated ROS was analyzed in the presence of specific ROS scavengers or quenchers. Thus, the quenching effect of 200 mM sodium azide on ^1^O_2_ or the scavenging of O_2_^•−^ by 200 mM Tiron on biofilm photoinactivation by PTN is shown in Figure 3. The addition of Tiron completely inhibited the photodynamic effect of PTN at all bioactive concentrations (pMIC and pMICx2). In contrast, sodium azide exhibited approximately half the efficacy of Tiron. Although it was unable to reverse the action of the O_2_^•−^, sodium azide significantly increased the survival of biofilms by almost three logs at these concentrations.

#### 3.2.2. Biofilm Stress Response After PTN-APDT

**Figure 4:**
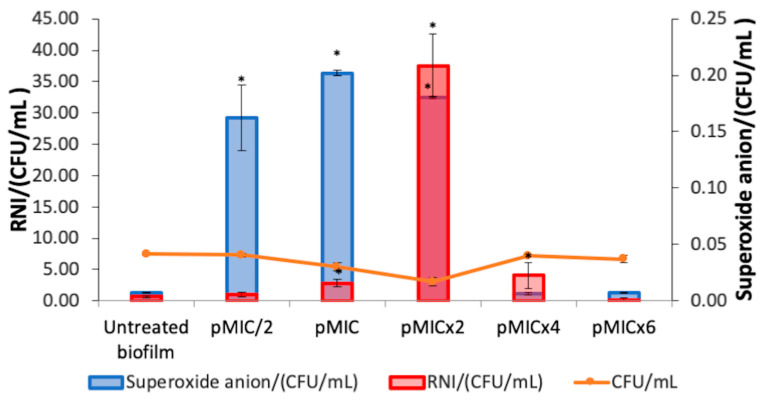
Reactive oxygen species (ROS) and reactive nitrogen intermediates (RNI) production on *C. tropicalis* biofilm treated with parietin and irradiation, using pMIC (0.98 μg/mL ≈ 3.45 μM) as reference. Data were obtained by the colony-forming unit (CFU) count test, corresponding to viable biofilm. * *p* < 0.05 with respect to *C. tropicalis*.

The O_2_^•−^ generation and nitrosative metabolite production were studied for the photoinactivation process of PTN on *C. tropicalis* biofilms. An increment in O_2_^•−^ levels was observed when PTN was photoexcited at pMIC/2, pMIC, and pMICx2 with respect to untreated biofilms (Figure 4, blue bars). In addition, at the higher PTN concentrations tested, no increase in this ROS was detected. In relation to RNI generation by this AQ when it was photostimulated on biofilms (Figure 4, red bars), the rise in RNI was greater for the most active concentration of PTN than the other concentrations tested.

**Figure 5:**
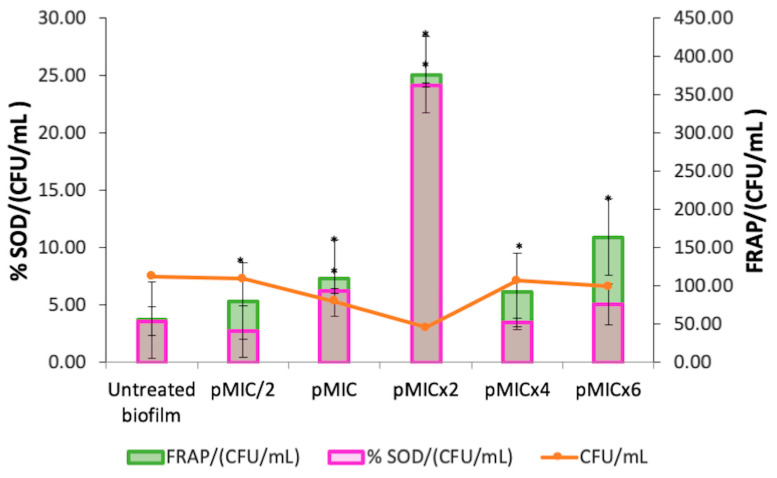
Activation of superoxide dismutase enzyme (SOD) and total antioxidant capacity (FRAP) in *C. tropicalis* biofilm treated with parietin and irradiation, using pMIC (0.98 μg/mL ≈ 3.45 μM) as reference. Data were obtained by the colony-forming unit (CFU) count test, corresponding to viable biofilm. * *p* < 0.05 compared to *C. tropicalis*.

Furthermore, the stimulation of SOD activity (Figure 5, magenta bars) was observed at the photoactive concentrations of PTN (pMIC and pMICx2). The levels of total antioxidant capacity (FRAP) in the treated biofilm increased at all conditions evaluated compared to the untreated biofilm (Figure 5, green bars). Moreover, both defense systems (SOD and FRAP) were detected even in higher proportion for pMICx2.

## 4. Discussion

The treatment of candidiasis is a multifaceted process, due to the pathogenic mechanisms of *Candida* spp., particularly their ability to form biofilms and their acquired resistance or multi-resistance, which represents a significant public health concern [3]. Given this challenge, aPDI is being investigated as a possible alternative for treating fungal infections, which was the motivation for this study [30].

In this study, we started by investigating the effect of PTN-mediated PDI on planktonic yeast of *C. tropicalis*. The results showed negligible toxicity in samples treated with AQ and darkness (<20% growth inhibition). This is consistent with a previous study that reported PTN induced low cytotoxicity in Vero cells. Furthermore, in this previous work, PTN had antibacterial effect on both Gram-positive and -negative strains only after photostimulation [16]. The effect of light-activated PTN against *C. tropicalis* and other clinically relevant fungal pathogens, such as *Candida albicans*, *Candida glabrata*, and *Candida auris*, has been described using a light-emitting diode (LED) irradiation system (λ = 428 nm). Although yeast growth inhibition was achieved at a lower concentration of PTN (0.156 μg/mL vs. 0.98 μg/mL), a significantly higher irradiation dose was required (30 J/cm^2^ vs. 0.59 J/cm^2^) [31]. Therefore, the lower irradiation dose employed was compensated by using a higher concentration of PS, without exhibiting cytotoxic effects on normal cells. Furthermore, the irradiation system employed in our study is widely used in phototherapy for the treatment of hyperbilirubinemia in humans (https://www.lighting.philips.es/prof/lamparas-y-tubos-convencionales/lamparas-especiales/diversas-aplicaciones-uv/PHOTHER_SU/category, accessed on 7 April 2025).

Similarly, Ma et al. investigated the in vitro PDI mediated by aloe-emodin on azole-sensitive and -resistant *C. albicans* planktonic yeasts [32]. After incubation with 10 μM aloe-emodin and irradiation with 96 J/cm^2^ of light, approximately 6.5 log^10^ reductions in the survival of azole-sensitive and -resistant *C. albicans* were achieved. Their findings further support the potential of anthraquinone derivatives as effective PSs against fungal pathogens, as they observed a significant reduction in yeast after light activation. In their study, a higher irradiation dose (96 J/cm^2^) was required to achieve substantial antifungal effects, in contrast to the lower doses employed in our work.

Our study also evaluated the PDI of an AQ on a *Candida* spp., but this was PTN on *C. tropicalis* biofilms. In the dark, PTN showed no antifungal effect at any of the concentrations tested (up to 5.9 μg/mL ≈ 20.7 μM). However, under actinic irradiation, the antibiofilm effect increased significantly, resulting in a 99.9% decrease in viability. Our results reveal that twice the concentration of PTN was required to produce the photodynamic effect in biofilms compared to planktonic growth. This is because biofilms are more resistant than their planktonic counterparts, as they are complex three-dimensional structures; however, they appear to favor the interaction of AQ, beginning the aggregation at a high concentration (pMICx4), as observed in Figure 3. This behavior has already been observed for other AQs (rubiadin and rubiadin-1-methyl ether) in previous studies [21]. Other natural PSs that align with the blue-light emission spectrum are curcumin and riboflavin. In this sense, Quishida et al. [33] investigated the PDI of curcumin and LED light against *C. albicans*, *C. glabrata*, and *Streptococcus mutans* biofilm. The findings revealed that aPDT significantly decreased biofilm viability, metabolic activity, and biomass. However, the photoantibiofilm effect required high concentrations of curcumin (80, 100, and 120 μM). Alshehri et al. [34] examined the effectiveness of PDI using 0.1% riboflavin on *C. albicans* biofilms. Blue LED light was utilized to activate riboflavin, and fungal cell viability was assessed using 3-(4,5-dimethylthiazol-2-yl)-2,5-diphenyltetrazolium bromide (MTT) assay. Treatment with riboflavin and LED resulted in the lowest survival rate of *C. albicans*, with viability below 50%.

Furthermore, other AQs such as emodin, alizarin, alizarin red, chrysazin, quinalizarin, purpurin, and 1-hydroxyanthra-9, 10-quinone have been shown to inhibit biofilm formation of *C. albicans*. The efficacy of emodin against *C. albicans* biofilm of strains isolated from hospitalized patients was achieved in the range of 200–400 μg/mL [35]. Alizarin and chrysazin reduced the metabolic activity of *C. albicans* biofilms by >98% at 10 μg/mL and by 66% at 2 μg/mL, respectively [36]. Moreover, inhibition of *C. albicans* biofilm formation by anthraquinones-related compounds, namely pyrocatechol, alizarin red, quinalizarin, emodin, 1-hydroxyanthra-9,10-quinone, purpurin, chrysazin, and alizarin has shown to be dose-dependent [35,37]. In addition, the antifungal activity of alizarin has been largely attributed to the presence of aromatic hydroxyl groups (-OH) in its AQ core [36]. This suggests that variations in the position of the -OH groups significantly influence the antibiofilm properties. Among the AQs tested, those with a -OH group at position-1(adjacent to the carbonyl group) exhibited the highest activity in all assays. Notably, purpurin, chrysazin, and alizarin, which share an -OH group at the C1 position, demonstrated strong inhibitory effects against *Candida albicans* biofilms even at low concentrations (2 μg/mL) [36]. Therefore, the antibiofilm activity of PTN against *C. tropicalis* may be influenced by the presence of the -OH group at position-1 in the AQ structure.

Besides the AQs, hypericin (a dianthrone) and porphyrins have emerged as promising PSs with high selectivity for fungal pathogens. Hypericin-mediated aPDT demonstrated significant antifungal activity, achieving substantial reductions in *Candida* biofilm and planktonic cell viability. Furthermore, hypericin activated by yellow or orange light effectively affected fluconazole-resistant *Candida* strains with minimal cytotoxicity to host tissues [38,39].

Porphyrins are PSs with high selectivity to pathogens. Thus, the photoinactivation of different *Candida* spp. by two cationic porphyrins derived from tetra-chloride salts, meso-tetra(4-N-methylpyridyl) porphyrin and meso-tetra(3-N-methylpyridyl)porphyrin derivatives (4-H2TMeP+ and 3-H2TMeP+), and anionic porphyrin tetra-sodium salt, meso-tetra(4-sulfonatophenyl)porphyrin (4-H2TPSP−) was investigated by Amorin et al. [40]. For the irradiation process, a white-light LED and light dosage of 10.9 J/cm^2^ was used. Cationic porphyrins can photoinactivate different *Candida* spp. in both planktonic and biofilm forms. *C. tropicalis* showed 35.8% interference when treated with 4-H2TMeP+, and 51.67% with 3-H2TMeP+ at 75uM for both porphyrins [40]. In addition, the tetracationic metalloporphyrin Zn(II) meso-tetrakis(N-n-hexylpyridinium-2-yl)porphyrin (ZnTnHex-2-PyP4+) was assayed against biofilms of *C. albicans* strains (ATCC 10231 and ATCC 90028) using a blue light-emitting diode. The PDI (4.3 J/cm^2^) at 0.8 μM showed about 89% decrease in the cell viability as assessed by the MTT assay [41].

Therefore, PTN stands out as an effective PS against *Candida tropicalis*, displaying strong antifungal and antibiofilm activity upon light activation, whereas remaining non-toxic in the dark. As an AQ-based compound, it benefits from a well-defined photoreactive core, structural stability, and required -OH positioning, which enhances its photodynamic efficacy. Its potent effect, even at low irradiation doses, makes it a promising alternative to other natural PSs that require higher concentrations or light doses for comparable activity.

The photoinactivation mechanism of *C. tropicalis* biofilms by PTN was investigated to identify the photoprocesses occurring in PTN-PDI.

Tiron inhibits the photoinactivation *C. tropicalis* biofilm by PTN at bioactive concentration. Although Tiron is a quencher of O_2_^•−^, it can react with hydroxyl radicals (HO^•^) [42]. Since HO^•^ production occurs after the O_2_^•−^ generation and Tiron was added before the irradiation period, it can be inferred that Tiron acted as a scavenger of O_2_^•−^. Thus, the Type I photosensitization mechanism would be mainly involved in PTN-mediated PDI in the biofilm. Furthermore, our previous work showed that PTN can produce O_2_^•−^, being an efficient Type I photosensitizer [16]. On the other hand, sodium azide only has a limited protective effect on *C. tropicalis* biofilm, despite PTN being an efficient ^1^O_2_ producer [16]. This type of behavior has also been reported for other AQs, such as rubiadin and its methylated derivative. For both AQs, it was shown that the main photosensitizing mechanism involved in the photoinduced antibiofilm activity is the O_2_^•−^ formation through suppression of AQ excited states by electron transfer, whereas the role of ^1^O_2_ seems to be less significant [25].

Oxidative stress is a complex process that occurs because of two main factors: an overproduction of ROS, specifically by O_2_^•−^ levels (Type I mechanism), given the short lifetime of singlet oxygen (3 μs) [43], and a reduction in oxidative defenses. Thus, this excessive ROS cannot be compensated by the enzymatic and non-enzymatic antioxidant systems of the biofilm, which make them insufficient to remove free radicals, resulting in damage to macromolecules (DNA, protein, lipids) and cellular components (cell membrane) [44,45].

When studying the biofilm response to this oxidative burst, NO generation should also be assessed as a response to O_2_^•−^ levels, since this occurs more rapidly than the enzymatic response of SOD, thus promoting biofilm photoinactivation [46]. Elevated levels of NO (µM concentration) can cause cytotoxicity by inducing oxidative and nitrosative stress, leading to DNA damage, enzyme modification, cellular dysfunction, inflammation, mitochondrial impairment, and apoptosis. NO can inhibit key mitochondrial enzymes, such as aconitase, and disrupt respiratory chain complexes. It also reacts with O_2_^•−^, at a faster rate than with oxygen, to form the toxic peroxynitrite (^−^OONO), which generates harmful radicals (HO^•^ and NO). It may react with H_2_O_2_ to produce ^1^O_2_, further intensifying damage [47,48].

Similar oxidative and nitrosative imbalances have been reported with other AQs, such as rubiadin and its 1-methyl ether derivative, isolated from *Heterophyllaea pustulata*. These compounds also induce significant photoreductions in biofilm formation of *C. tropicalis*, likely through similar mechanisms involving the overproduction of ROS and RNI [24].

In planktonic cells, oxidative stress induced by ROS overproduction has been extensively studied. Fiala et al. (2024) [31], showed that PTN targets the cell membrane and induces cell death via ROS-mediated lipid peroxidation after light irradiation on *Candida* planktonic yeast. Aloe-emodin has also been reported to induce photodynamic damage to the cell envelope of *C. albicans* planktonic form through ROS generation [32].

All this background suggests that the oxidative burst, resulting from ROS and RNI, is a common feature in the photoantimicrobial action of AQs, as our results also show.

The study of natural PSs contributes to the development of aPDT, which has proven to be selective, fast acting and has not developed resistance to date. This selectivity is achieved through strict spatial control of irradiation on the affected area (infection), thus uniting the three agents responsible for the photodynamic effect (PS, light, and ^3^O_2_). The PSs used in aPDT have not been shown to stimulate resistance mechanisms in microorganisms due to their multidimensional action, which generates a generalized oxidative imbalance that does not differentiate between resistant and non-resistant microorganisms. This photooxidation even includes the destruction of virulence factors, which are generally organic molecules susceptible to oxidation. Moreover, the PSs do not necessarily need to enter the cell to exert its action. Consequently, the pathogen cannot develop resistance through increased detoxification metabolism or export of the drug [49].

## 5. Conclusions

In conclusion, this study demonstrates that the photoinactivation of *Candida tropicalis* biofilms using PTN as the photosensitizer is primarily driven by O_2_^•−^ (Type I mechanism), with ^1^O_2_ (Type II mechanism) playing a secondary role. However, the photodymamic effect is dependent on the concentration of PTN, since at higher concentration PTN self-aggregation effects play a role in the prompt deactivation of the excited states of the AQ. The results also show an imbalance in NO levels, which likely contributes to the cellular stress response and enhances the effectiveness of the treatment. The dominant role of O_2_^•−^ in biofilm inactivation highlights its significance in the photodynamic mechanism of PTN. These findings suggest that PTN ability to disrupt fungal biofilms is linked to complex oxidative and nitrosative stress pathways, making PTN a promising candidate for antifungal PDT and potential clinical applications.

## Data Availability

The original contributions presented in this study are included in the article. Further inquiries can be directed to the corresponding author.

## Abbreviations

The following abbreviations are used in this manuscript:

Φ_Δ_	Quantum yield of singlet oxygen
+C	Positive control
^1^O_2_	Singlet oxygen
^3^O_2_	Molecular oxygen
AmB	Amphotericin B
aPDI	Antimicrobial photodynamic inactivation
aPDT	Antimicrobial photodynamic therapy
AQ/s	Anthraquinone/s
ATM	Antimicrobial
CFU	Colony-forming units
CLSI	Clinical and Laboratory Standards Institute
EtOH	Ethanol
FeSO_4_	Ferrous sulfate
FRAP	Ferrous Reduction Antioxidant Potency assay
H_2_O_2_	Hydrogen peroxide
HO^•^	Hydroxyl radical
MIC	Minimal inhibitory concentration
MOPS	Morpholine propane sulfonic acid
NaN_3_	Sodium azide
NaNO_2_	Sodium nitrate
NaOH	Sodium hydroxide
NBT	Nitro-blue tetrazolium
NCPF	National Collection of Pathogenic Fungi
NO	Nitric oxide
O_2_^•−^	Superoxide radical anion
OD	Optical density
PBS	Phosphate buffered saline
pMIC	Photoactive minimal inhibitory concentration
PS	Photosensitizer
PTN	Parietin
RNI	Reactive nitrogen intermediates
ROS	Reactive oxygen species
RPMI	Roswell Park Memorial Institute 1640
SDA	Sabouraud dextrose agar
SDB	Sabouraud dextrose broth
SOD	Superoxide dismutase
MFC	Minimum fungicidal concentration
MTT	3-(4,5-dimethylthiazol-2-yl)-2,5-diphenyltetrazolium bromide
-OH	Hydroxyl groups
DNA	Deoxyribonucleic acid
^−^OONO^−^	Peroxynitrite
